# Optimizing Complexity Measures for fMRI Data: Algorithm, Artifact, and Sensitivity

**DOI:** 10.1371/journal.pone.0063448

**Published:** 2013-05-21

**Authors:** Denis Rubin, Tomer Fekete, Lilianne R. Mujica-Parodi

**Affiliations:** 1 Department of Applied Mathematics and Statistics, State University of New York at Stony Brook, Stony Brook, New York, United States of America; 2 Department of Biomedical Engineering, State University of New York at Stony Brook, Stony Brook, New York, United States of America; Universidad Veracruzana, Mexico

## Abstract

**Introduction:**

Complexity in the brain has been well-documented at both neuronal and hemodynamic scales, with increasing evidence supporting its use in sensitively differentiating between mental states and disorders. However, application of complexity measures to fMRI time-series, which are short, sparse, and have low signal/noise, requires careful modality-specific optimization.

**Methods:**

Here we use both simulated and real data to address two fundamental issues: choice of algorithm and degree/type of signal processing. Methods were evaluated with regard to resilience to acquisition artifacts common to fMRI as well as detection sensitivity. Detection sensitivity was quantified in terms of grey-white matter contrast and overlap with activation. We additionally investigated the variation of complexity with activation and emotional content, optimal task length, and the degree to which results scaled with scanner using the same paradigm with two 3T magnets made by different manufacturers. Methods for evaluating complexity were: *power spectrum*, *structure function*, *wavelet decomposition*, *second derivative*, *rescaled range*, *Higuchi’s estimate of fractal dimension*, *aggregated variance,* and *detrended fluctuation analysis*. To permit direct comparison across methods, all results were normalized to Hurst exponents.

**Results:**

Power-spectrum, Higuchi’s fractal dimension, and generalized Hurst exponent based estimates were most successful by all criteria; the poorest-performing measures were wavelet, detrended fluctuation analysis, aggregated variance, and rescaled range.

**Conclusions:**

Functional MRI data have artifacts that interact with complexity calculations in nontrivially distinct ways compared to other physiological data (such as EKG, EEG) for which these measures are typically used. Our results clearly demonstrate that decisions regarding choice of algorithm, signal processing, time-series length, and scanner have a significant impact on the reliability and sensitivity of complexity estimates.

## Introduction

The prevalence of power-law or scale-free behavior in natural processes is well-established [1], with theoretical justification for the complexity found in neurobiological and physiological systems [2], [3]. These include considerations of robustness, in which redundancies maximize system integrity in the event of damage, as well as adaptability: operating on the edge of chaos, complex systems position themselves for optimal responsivity to inputs, as well as ability to maintain homeostatic regulation. The complexity of brain activity has been observed and modeled on many levels, from neuronal spiking [4], [5] and local field potentials [6] to EEG [7], suggesting that scale-free behavior may be fundamental to neural information processing. Recent fMRI experiments demonstrate that brain signal fluctuations differ from other scale-free natural processes, suggestive of different underlying mechanisms [8], and that fMRI time series exhibit complexity that is functionally significant [9]. Moreover, there is evidence that deviations from chaotic behavior can be used diagnostically in identifying disease, using ECG [10]–[12], EEG [13], MEG [14], NIRS [15], and fMRI [8], [16]–[18].

FMRI applications of complexity have dealt with both between-voxel and between-subject differences. For example, active and inactive voxels in the human visual cortex show markedly different power-law and Hurst exponents during a simple rotating checkerboard fMRI paradigm [19]. Shimizu et al. [20] applied a multifractal version of the Hurst exponent (the Hölder exponent) to fMRI data to show that active voxels are clearly distinct from both non-active ones and white matter. More recent results illustrate that the power law exponent varies across networks of voxels (e.g. attention, default, motor, saliency, and visual) [8] and is affected by cognitive load [21]; for review, see [22] and [3]. Between-group differences in time series complexity either focused on a particular disorder, such as Alzheimer’s disease [17], autism [16], and schizophrenia [18], or individual variability across psychophysiological variables such as trait anxiety, heart rate variability [23], age, cholinergic effects, and cognitive performance [24], [25].

Complexity measures in physiology have historically been applied to time-series that are long, rich, and have strong signal/noise, as with 24-hour ECG. In contrast, fMRI time-series tend to be short (5–10 minutes), sparse (TR = 1–2.5 s), and subject to sufficient scanner and physiological artifacts that significant pre-processing is the norm. Despite the application of complexity methods to fMRI data, and the challenges associated with it, no systematic analyses of the optimal way to compute such complexity measures specifically for fMRI data have been published. To this end, here we evaluate the most common methods for computing time series complexity, investigate the effects of data preprocessing, activation and scanner differences, with eye towards optimizing the balance between detection sensitivity and resilience to artifact. For the sake of permitting direct comparison between methods, each of which is scaled differently, all values will be normalized to the Hurst exponent.

## Methods

### 2.1 Overview

This paper references a range of fMRI datasets, with all data collected on a single Siemens Magnetom Trio 3T scanner, unless specified otherwise. The tasks include a resting state design (RST; N = 11), a “guided-rest” design modified to standardize content across subjects by presentation of a movie, the pilot episode of the television series “Lost” (GRST-LOST; N = 11), event-related presentation of emotionally valent faces (ER-FACES; N = 22), block presentation of faces (BL-FACES; N = 22), and a block design auditory anticipation task (BL-ANT; N = 22). The GRST-LOST task was implemented mainly to provide a realistic design-free stimulus that would ensure that participants do not fall asleep during the lengthy scan session designed to provide long time series. The particular choice of show was made based on the general public consensus that the show is highly rated (IMDB rating of 9.3) and therefore would be engaging. The other tasks (BL-ANT, ER-FACES, BL-FACES) were chosen for the purpose of task design comparison, as all three sets of stimuli were presented to the same 22 participants.

### 2.2 fMRI Acquisition Parameters

For the three sets of stimuli presented to the same 22 participants (BL-ANT, ER-FACES, BL-FACES), we acquired fMRI data using a 3 Tesla Siemens Trio whole body scanner equipped with an eight-channel SENSE head coil. We acquired T2*-weighted whole-brain volumes with an EPI sequence with the following parameters: TR = 2500 ms, TE = 22 ms, flip angle = 83°, matrix dimensions = 96×96, FOV = 224 mm×224 mm, slices = 36, slice thickness = 3.5 mm, gap = 0. For RST and GRST-LOST the TR was changed to 2100 ms and the number of slices to 37. Standard preprocessing procedures were performed in SPM8, including image realignment corrections for head movements, slice timing corrections for acquisition order, normalization to standard 2×2×2 mm Montreal Neurological Institute space, and spatial smoothing with a 6-mm full width at half maximum Gaussian kernel. The BL-ANT task was preprocessed in SPM5, but the procedures used produce numerically identical results in SPM5 and SPM8. As for the other tasks, the standard preprocessing in SPM8 included realignment, normalization, and spatial smoothing with the same parameters as above. The number of acquired volumes in these tasks was as follows: BL-ANT = 232, ER-FACES = 226, BL-FACES = 264, RST = 143, GRST-LOST = 1190.

### 2.3. Task Description

The GRST-LOST data were collected during an uninterrupted scanning session, with a five-minute pure rest condition (RST) always preceding guided-rest condition (GRST-LOST). During the rest condition, participants were instructed to relax and let their minds wander while looking at a fixation cross and waiting for the show to start. Immediately after the rest condition, participants viewed the entire first part (42 minutes) of the pilot of ABC’s TV-series “Lost” (GRST-LOST). Using simultaneous eye-tracking during scans, we verified that all participants stayed awake during the entire course of the experiment. Immediately after watching the episode, the participants rated the content of the show on a nine-point scale in terms of arousal (“excitement”) and valence (“pleasantness”). The ratings were provided for each of the two hundred 10–15 second scenes into which the episode was split. The participants were instructed to rate their emotional responses rather than the content. The sequences of clip ratings were interpolated to match the length of the show and averaged across subjects. The valence scores were inverted for ease of interpretation.

In the BL-ANT task, the participants anticipated an unpleasant or a neutral stimulus during a 16-second countdown preceded by a cue informing them of the upcoming stimulus. The stimuli were aversive (loud) or benign (soft) bursts of white noise. A complete description of the anticipation task and its findings using standard (GLM) analyses were previously published [26].

For the BL-FACES task, the Ekman faces [27] were presented in 20-second blocks of nine faces, each block containing either angry, fearful, neutral, or happy faces. The sequence of blocks was counterbalanced for order across participants. This design was equivalent to one used in our previous studies [18], [23]. The ER-FACES task required more stimuli for a comparable length of time, so we used a subset of Karolinska faces [28], with fearful and happy emotional expressions (26 individuals, 13 female). The images were cropped along the hairline and converted to grayscale. The design was generated using OptSeq [29] using 2–10 second inter-stimulus intervals.

### 2.4. Complexity Estimation and Normalization to Hurst Exponent

The generalized Hurst exponent was computed for time series *x(t)* for *q* = 1 and *q* = 2 according to the structure function: 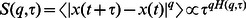 [30]. For time series of length *L*, we used ten logarithmically spaced lags *τ* ranging between 1 and *log*_10_(*L*/50). The estimates of *H(q)*, denoted by *H_Q1a_* and *H_Q2a_*, were estimated by the slope of line fitting log(*S*(*q,τ*))*/q* vs. log(*τ*).

The generalized Hurst exponent was also estimated by a different implementation [31] of the same relation in accordance to [32]–[34]. Again, the Hurst parameter was estimated for *q* = 1 and *q* = 2 (*H_Q1b_/H_Q2b_*), with the default maximum lag of 19.

An alternative method for estimating Hurst exponent is the aggregated variance technique [35], available through Matlab File Exchange [36], [37]. A series of length *N* is divided into *N*/*m* blocks of length *m*, and sample variance of each block *X^(m)^* is computed. For series of finite variance, the sample variance *var(X^(m)^)* will be asymptotically proportional to *m* ^2*H*-2^ for large *N*/*m* and *m*. The Hurst exponent *H_AV_* is estimated by computing the slope 2*H_AV_*-2 of the graph of sample variance vs. *m*.

Another closely related quantity is Higuchi fractal dimension [38], which estimates the fractal dimension *D* of a time series curve from segments *L(k)* constructed from *k* samples, with 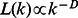, where *D* is related to Hurst estimate *H_HFD_* through *D = *2*–HFD* [39]. We used *k* = 5 and *k* = 10 samples, denoted by *H_HFD-5_* and *H_HFD-10_*, respectively. A Matlab implementation of the algorithm [40] can be found through Matlab File Exchange [41].

The Matlab Wavelet Toolbox [42] also provides several estimates of the Hurst parameter. The first two estimates are based on the discrete second-order derivative, using an FIR and a wavelet filter, denoted *H_DD_* and *H_DDW_*, respectively [43]. The third estimate is obtained from the slope of the variance of local detail coefficients vs. scale [39]. For the last estimate, in addition to the default Haar wavelet, we used higher order Daubechies wavelets of scales 2, 4, 8, and 16, denoted by *H_db1_*, *H_db2_*, *H_db4_*, *H_db8_*, and *H_db16_*.

The Hurst parameter can, under certain assumptions [44], be computed through the relation *H* = (*β*–1)/2, where *β* is the negative slope of the power spectral density of the time series *PSD(x)* vs. frequency *f* on a log-log scale (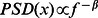). We used two methods to estimate the power spectrum; one employing the Matlab fast Fourier transform (*PSD*(*x*) = |*FFT*(*x*)|^2^) and the other used Welch’s periodogram with eight windows of 50% overlap. The corresponding values of the Hurst exponent calculated from *β* estimates are denoted *H_FFT_* and *H_pWelch_*, respectively.

The estimation of the Hurst parameter via Detrended Fluctuation Analysis (*H_DFA_*) was performed in accordance with [11]. First, each *N*-point long time series *x*(*t*), *t* = 1. *N*, is integrated 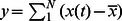 and a set of window – or box – lengths is defined. We chose box sizes on a log-scale between 4 and *N*/4 points such that the ratio between successive box sizes was 2^1/8^. For each box size, time series segments of that many points are created by a sliding window method. A linear trend is fit and subtracted from each segment and the root-mean-square of the residuals is computed. More formally, for each window length *n* and set of *k* segments *y*(*k*), the root-mean-square fluctuation is 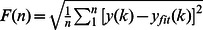, where *y_fit_*(*k*) is the linear fit to the segment *y*(*k*). This calculation is repeated for each box size *n* and the slope of the log-log scale fit of *F*(*n*) vs. box size yields the scaling exponent *α*, where *H* = *α*–1 [2]. Since the DFA estimate is affected by the range of box sizes, we also computed DFA over small and large box sizes (*H_DFA-S_/H_DFA-L_*) by fitting the upper and lower parts of *F*(*n*) vs. box size separately. For example, for our longest 1190-point time series, small boxes ranged 4–40 points and large ranged 40–300 points).

*H* via Rescaled Range was estimated using the relationship 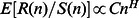
_,_ where *S(n)* is the standard deviation and *R(n)* is the range of cumulative sums of zero-meaned *n*-size segments of time series *X_i_*, *i* = 1.*L*. The recipe for computing is as follows. (1) Define a range of scales: *L,L/2,L/4,…L/k* such that *L/k* > = 8. (2) For each scale, define *n*-length segments of time series (e.g. one segment for scale *L*, two for *L/2*, etc.). (3) For each segment of length *n*, subtract the mean and compute cumulative sum 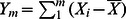, with *m* = 1.*n*. (4) Calculate *R(n)* = max(*Y*_1_.*Y*_n_) – min(*Y*_1_.*Y*_n_) over standard deviation *S(n)*. (5) Find E[*R/S*] by averaging *R/S* over all segments for a particular scale. (6) Estimate Hurst exponent *H_RS_* as the slope of log_2_(E[*R/S*]) vs. log_2_(scale) (e.g. |log_2_(*L*)|, |log_2_(*L*/2)|, etc.).

In order to test the consistency of measures, we generated 1000 time series with the *wfbm* function for wavelet-based generation of fractional Brownian motion with *H* <$>\raster="rg1"<$> [0,1] (Matlab’s Wavelet Toolbox) and compared the estimated values of *H* with the generated ones. Robustness of each measure was assessed through introducing spikes by changing values of a random subset of points within each of 1000 time series generated with *H* = 0.5. The magnitude of spikes ranged from zero to six standard deviations of the simulated time series. Spikes were both positive and negative in magnitude. The number of spikes varied from zero to five percent of the time series length. The susceptibility to spikes was estimated as a t-statistic describing the change in estimated value of the Hurst exponent as compared to the estimate of *H* from time series without spikes.

### 2.5. Correlation between Measures

Since all of the methods attempt to estimate the same quantity, we assessed their consistency by applying the measures to the same time series. Measure precision – the consistency of estimating a particular Hurst exponent – was estimated by correlating Hurst estimates for different measures applied to the time series with *H* = 0.5 (*N* = 1000). Measure accuracy – the ability to estimate a range of Hurst exponents accurately – was assessed by correlating Hurst estimates for different measures across 1000 time series with Hurst exponent ranging from zero to one.

### 2.6. Error Drop-off

The error of the estimates σ decreases as the length of the time series L increases, which can be expressed by the relationship 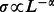, where *α* is the error drop-off rate. *α* was computed for “brown noise” time series of ten lengths ranging from 100 to 10000 points over 100 instances.

### 2.7. Signal Processing

Various signal processing techniques are applied to fMRI time series in order to improve signal to noise ratio. For example, it is commonly accepted that high-pass filtering and inclusion of motion parameters in the regression model is appropriate for most GLM analyses. Other techniques involve inclusion of higher order motion terms to account for nonlinearity of magnetic field distortion. In resting state connectivity literature, band-pass filtering is often applied to exclude fluctuations beyond a certain frequency, since they are believed to be non-physiological. Inter-subject correlation analyses suggest removal of global signal to avoid inflation of correlation values [45]. However, to the best of our knowledge, no comprehensive comparison of effects of signal processing on complexity of fMRI time series has been performed to date.

The types of corrections applied to the time series were detrending (denoted by ‘d’), regressing out motion parameters (denoted by ‘m’) and their squares (denoted by ‘2’), high-pass filtering at 0.01 Hz (denoted by ‘fhi’), band-pass filtering in the 0.01–0.1 Hz range (denoted by ‘fbp’), and regression of the global signal out of the time series (denoted by ‘bcw’), which consisted of three regressors: mean time series of the whole brain, cerebrospinal fluid, and white matter. The motion parameters were derived from SPM’s realignment procedure, the filtering was performed using 10^th^ order Butterworth filter, and global regressors were obtained using canonical masks for brain, white matter, and cerebrospinal fluid included in the SPM8. We considered all reasonable combinations of corrections, excluding higher order motion correction ‘2’ without the linear term and redundancy of ‘fhi-fbp’ combination, which resulted in 36 combinations that included the original untouched time series (denoted by ‘o’).

The success of the correction was assessed in two ways: by comparison of values Hurst estimates within grey and white matter voxels and by strength of relation to activation measured as the area under the curve (AUC) of ROC curves constructed using SPM’s t-map of activation in response to *task>rest* contrast. The true positives were voxels with top 1% of the *t*-values – a criterion ensuring a constant ratio of true to false positives across subjects, regardless of strength of the individual response to the task. As an alternative measure of activation to the task we also computed cross-subject, or inter-subject, correlations (ISC) that showed which time series co-varied across subjects throughout the task. The cross-subject correlations were computed via pairwise correlations between subjects’ time series (for each voxel, after detrending, regressing out the motion and global signal, and high-pass filtering), converting correlations to *z*-scores using Fisher’s transformation, and averaging across subjects [46]. The ISC, designed to pick up activation to GRST-LOST, allowed us to compare this task to BL-FACES and ER-FACES.

## Results

### 3.1. Measure Error and Susceptibility to Spikes (Simulated Data)

**Figure 1:**
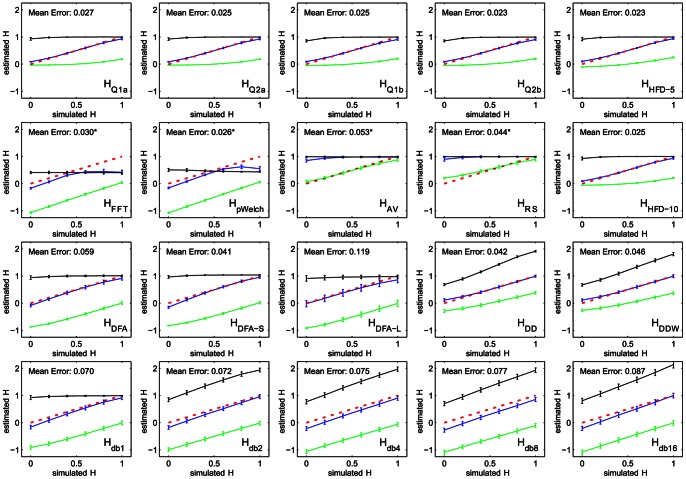
Estimates of the Hurst exponent are consistent and monotone for simulated wavelet-based fractional Brownian motion time series with Hurst exponent ranging from zero to one. Along with estimates for the simulated time series (blue), estimates for the time series integral (black) and derivative (green) are also shown. The red dotted line represents the theoretical value. Mean error is calculated as the average of the errors at each point; starred errors were computed for the derivative.

As **Figure 1** illustrates, most measures return the same values of *H* that were used to generate the time series. The few exceptions are *H_AV_*, *H_RS_*, and power spectrum based measures, *H_FFT_* and *H_pWelch_*. For *H_AV_* and *H_RS_*, a derivative needs to be applied to transform fractional Brownian motion (fBm) to fractional Gaussian noise (fGn). *H_FFT_* and *H_pWelch_* are not intrinsically bounded at 0.5 since there is no natural upper limit on the slope of the log-power-spectrum: rather the upper bound of the spectrum exists due to the way wavelet-based fBm time series are generated. We therefore used the derivative offset by one to obtain *H* estimates for simulated data.

The Hurst estimates of the integral and the derivative of the time series show that the majority of the measures are bounded from above and below, which means that *H*-estimates are artificially squeezed within the theoretically valid range for the Hurst exponent – a potential loss of sensitivity in case experimental data exhibits unusually high or low complexity. The unbounded measures – *H_FFT_*, *H_pWelch_*, H_db*_(except *H_db1_*), *H_DD_*, *H_DDW_* – show an approximately constant offset as a result of time series integration and differentiation. The average error on the *H*-estimates was on the order of ∼0.05 and consistent over the [0,1] range, with *H_DFA-L_* and H_db*_ showing the largest errors.

**Figure 2:**
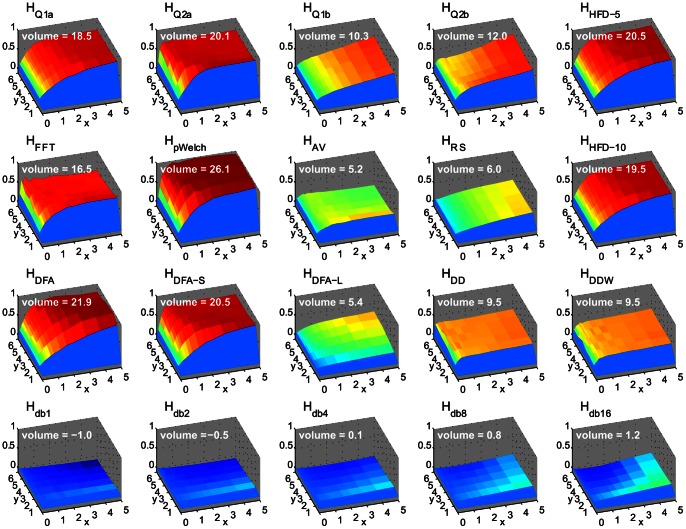
Number of spikes (as a percentage of points in a time series, x-axis) and their magnitude (in units of standard deviation of original time series, y-axis) affect Hurst estimates differently. The z-axis represents normalized to [0, 1] range one-sample t-test differences from the *H*-estimates of time series without spikes (each point on the surface is the t-value difference in *H* resulting from the introduction of spikes to the time series; the set of all t-scores for all measures was linearly transformed to [0, 1] range to show relative change due to presence of spikes). Volume is calculated as the difference between the surface and the plane defined by *H*-values of unaltered time series (spike magnitude of zero).

**Figure 2** illustrates the susceptibility of the measures to spikes as a function of their number and magnitude. As expected, all measures gravitated toward “white noise” values as the number of spikes and their magnitude increases. *H_Q2a_*, the second derivative estimates (*H_DD_*, *H_DDW_*) and FFT-based estimates (*H_FFT_*, *H_pWelch_*) were most sensitive to small number of spikes, reaching an upper bound quicker than other measures. H_Q*b_ and *H_RS_* showed an almost uniform increase regardless of spike amplitude. H_db*_ estimates were least susceptible to spikes, but low-order H_db*_ showed slight decreases in *H* as the number and magnitude of spikes increased.

### 3.2. Correlation between Measures (Simulated Data)

**Figure 3:**
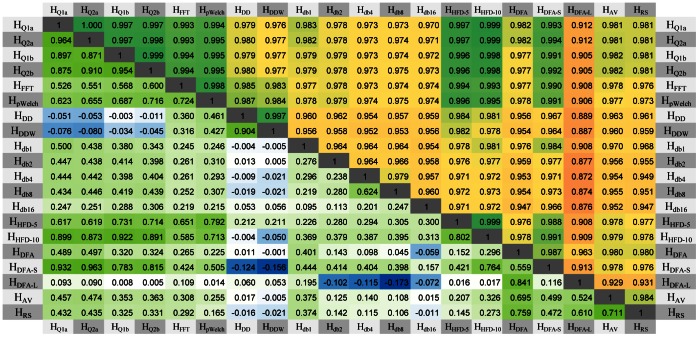
Correlations between Hurst estimates of simulated time series. Correlations between Hurst parameter estimates show that they are consistent but not precise. *Below Diagonal*: correlations between the measures computed over the same 1000 time series with *H* = 0.5 show that for most measures, errors on estimating the same exponent are not strongly correlated (light green to white to blue) with the exception of a few positively correlated (dark green) measures. *Above Diagonal*: correlations between Hurst estimates of the same 1000 time series with *H* values distributed uniformly between 0 and 1 show that measure estimates are consistent across a range of exponents, with some measures showing stronger correlation (green) than others (yellow).

The correlations between Hurst estimates of the same 1000 time series with *H* values distributed uniformly between 0 and 1 were very high, indicating reliability (**Figure 3**, values above diagonal). Among the least correlated measures were H_db*_, *H_DFA_*, *H_DFA-L_*, *H_AV_*, and *H_RS_*. Although most measures produced accurate estimates of the Hurst exponent, the correlation between the measures computed over the same data with a specific exponent were significantly smaller (**Figure 3**, values below diagonal show the correlations for *H* = 0.5). The highest correlations occurred between H_Q*_, *H_FFT_*, *H_pWelch_*, H_HFD*_, and *H_DFA-S_*, and between *H_AV_*, *H_RS_*, and *H_DFA-L_*. *H_DD_* and *H_DDW_* were mostly correlated to each other and H_db*_ were only weakly correlated to each other and to other measures. These groupings reflect similarities in computation between various measures despite the markedly different algorithms used to compute the quantity of interest.

### 3.3. Decrease in Estimation Error with Time-series Length (Simulated Data)

**Figure 4:**
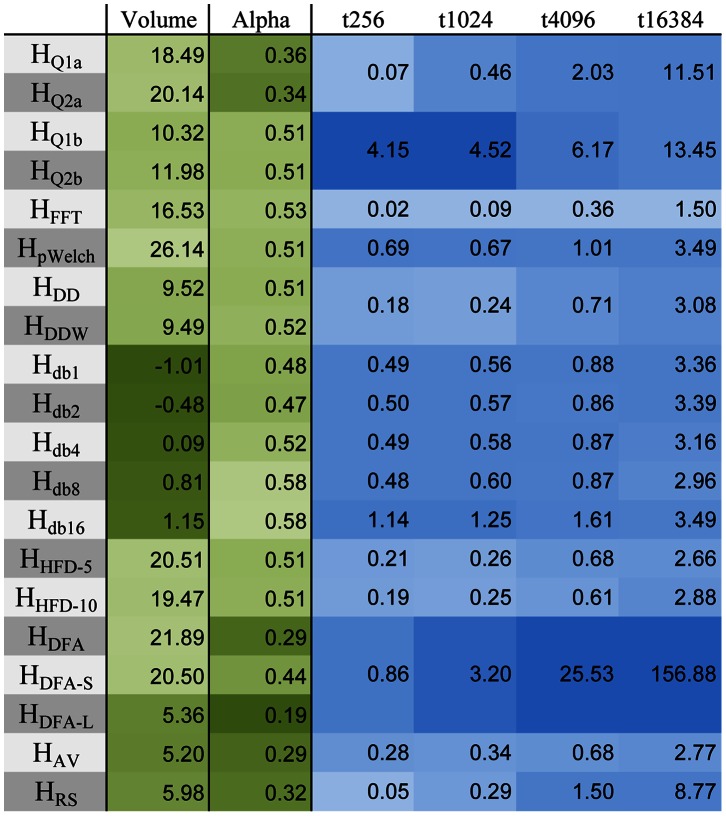
Comparison of Hurst estimates of simulated data. Volume under the surface of **Figure 2**, error drop-off rate alpha of **Figure 5**, and computation time (in seconds) for 1000 time series of length 256, 1024, 4096, and 16384 points. The computations, optimized using MATLAB Parallel Computing Toolbox, were performed on a quad-core Intel i7-960 @ 3.6 GHz.

**Figure 5:**
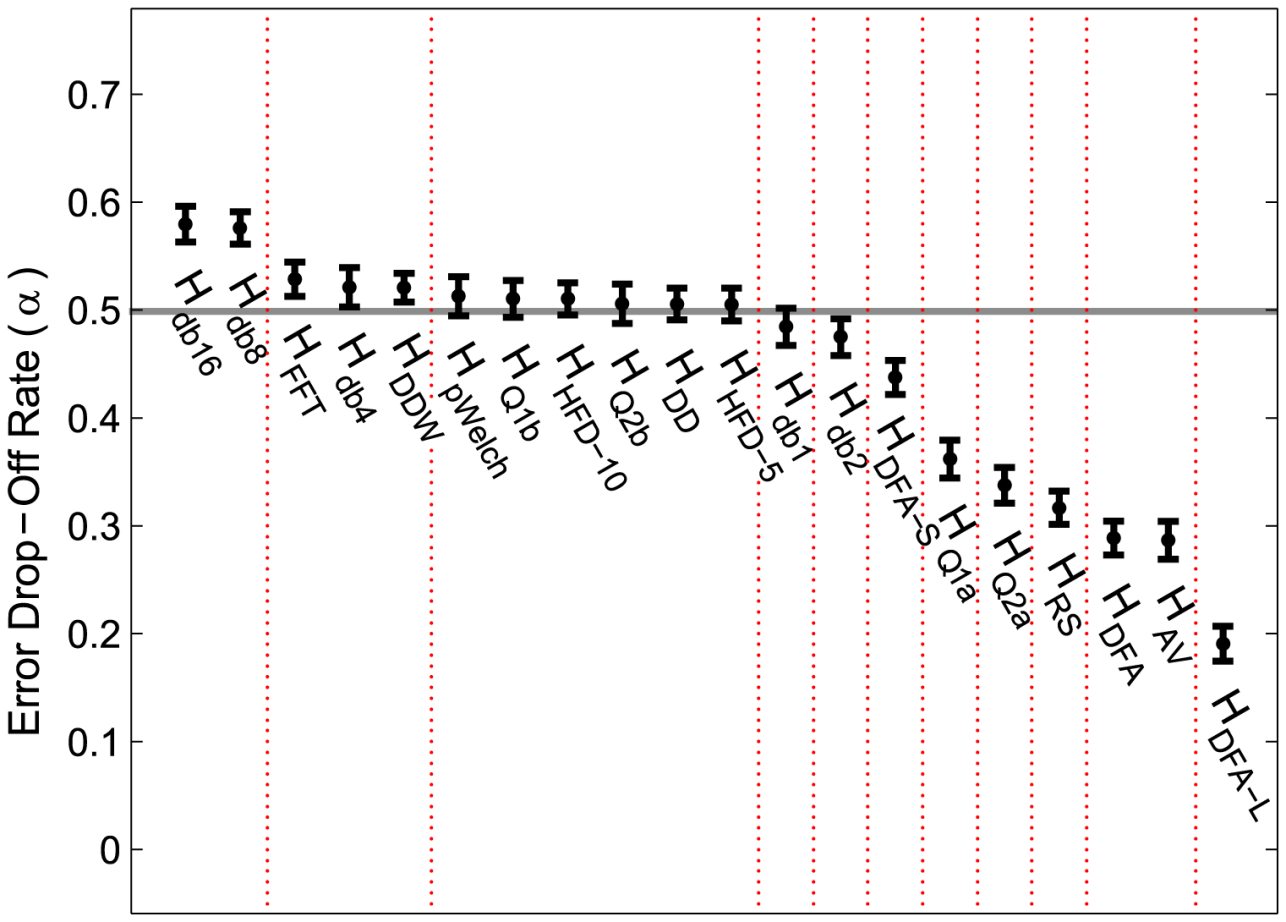
The complexity measures sorted by the average error drop-off rate *α* mostly follow the standard one over square root relationship (*α* = 0.5). The error drop-off rate was computed for “brown noise” time series of ten lengths ranging from 100 to 10000 points over 100 instances. The dotted vertical lines indicate which pairs of neighboring measures are significantly different at *p*<0.001 (uncorrected).

The error drop-off rate *α*, which is the rate of decrease in variance of the *H*-estimates as the length of the time series increases, is reported in **Figure 4**. For most measures, *α* is ∼0.5, which indicates a standard one over square root relationship, except for H_Q*a_, H_DFA*_, *H_AV_*, and *H_RS_*, which are less efficient (**Figure 5**).

The computation times for time series of varying length are shown in **Figure 4** as well. Although computation times depend on length and parallelizability of the implemented algorithms, the measures can roughly be separated into two categories – the ones whose compute times were within an order of magnitude of the fastest one and the ones that were not. The most efficient ones were H_Q*a_, H_DD*_, H_HFD*_, *H_FFT_*, *H_AV_*, and *H_RS_*. The time to compute Hurst estimate for an entire brain for a single person varied from minutes to hours depending on the choice of measure, with the overall computation time of 1.5–2 hours per person for all measures combined. Most of that time was taken up by the slower measures (H_Q*b_, H_db*_, *H_pWelch_*, and H_DFA*_), which renders these measures less practical for exploratory fMRI analysis.

### 3.4. Effect of Signal Processing: Detrending; Regressing Out Motion Parameters and Global Mean; Filtering (fMRI Data)

**Figure 6:**
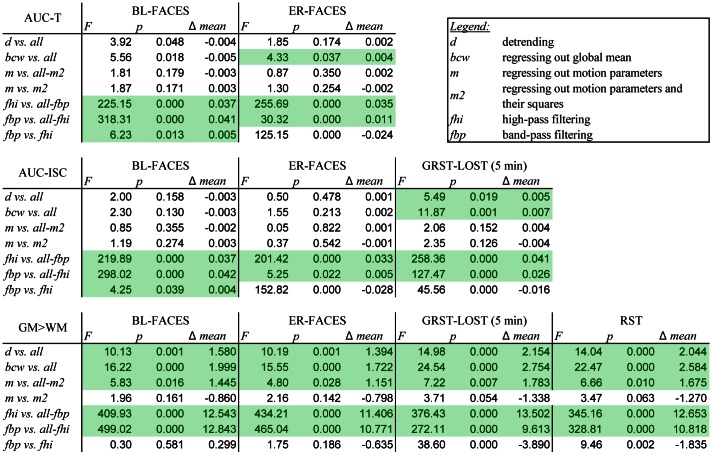
Comparison of combinations of signal processing. Results of an ANOVA of processing by measure (20) by subject (22 for FACES, 11 for GRST-LOST) show significant improvements in sensitivity and grey-white matter contrast of Hurst estimates (*df* = 1 for each comparison). The AUC-T values were derived from ROC curves constructed using top 1% of t-values of task vs. rest contrast. The AUC-ISC values were derived from ROC curves constructed using top 1% of z-transformed inter-subject correlations (ISC). The GM>WM values were derived from differences in Hurst estimates for grey and white matter voxels. Positive results significant at p<0.05 are highlighted.

We evaluated the effects of signal processing on Hurst estimates in terms of improvement of sensitivity to tissue type (assessed by grey-to-white matter contrast) and activation (assessed by AUC of ROC curves). Since the types of signal processing applied to the time series are not independent, we looked at the effect of adding a particular type of processing on top of all others. For example, to evaluate the effect of detrending, we compared all types of processing that involved linear detrending (12 types) to the ones that did not (24 types). Similarly, to evaluate the effect of high-pass filtering, we compared all types that involved high-pass filtering (12 types) to ones that were not high- or band-pass filtered (12 types). The results of comparisons are summarized in **Figure 6**.

In terms of increasing sensitivity to activation for the FACES tasks, for both t-contrast based and ISC based AUC values were most improved by high-pass filtering, which was significantly better than band-pass filtering for event-related design and only marginally worse than band-pass filtering for blocked design, suggesting that activation is best captured by dynamics above a certain frequency. For GRST-LOST, we used a five-minute segment three minutes into the show (GRST-LOST5) in order to make comparisons to other, shorter, tasks meaningful. For GRST-LOST5, both detrending, regressing out the global mean, and filtering increased AUC, but high-pass filtering still had the greatest effect. The agreement between t-value and ISC based results was not surprising, as there was over 60% spatial overlap for the top 3% of the values at the group level (66.5% for BL-FACES, 63.2% for ER-FACES). High-pass filtering remained the dominant effect for these three tasks as well as for RST task in terms of grey-white matter contrast, although both detrending and regressing out motion and global signal helped too. Second-order motion correction did not improve any of the metrics except GRST-LOST5, where it had the weakest effect.

In order to establish the best combination of processing steps, we computed contrasts for every combination vs. unprocessed estimates. For GM>WM contrast the clear winner for BL-FACES, ER-FACES, GSRT-LOST5, and RST tasks, was the *d-m2-bcw-fbp* combination (*Wald χ* ^2^ = 251.5, 208.4, 121.9, 152.3, respectively, *df* = 1, *p*<<0.0001), followed by the *d-m-bcw-fbp* combination (*Wald χ* ^2^ = 160.0, 122.6, 74.8, 92.9, respectively, *df* = 1, *p*<<0.0001) and the *d-m-bcw-fhi* combination (*Wald χ* ^2^ = 119.4, 107.1, 91.5, 87.8, respectively, *df* = 1, *p*<<0.0001). The contrasts were consistent for all tasks, with minimum correlation across processing combinations of 0.91. The combinations that gave the biggest improvements in the AUC-T and AUC-ISC values over the unprocessed time series involved high-pass and not band-pass filtering for ER-FACES and GRST-LOST5; the winner combination was *d-m2-bcw-fhi* (AUC-ISC *Wald χ* ^2^ = 29.5, 63.6, respectively, *df* = 1, *p*<<0.001) followed closely by *d-m-bcw-fhi* (AUC-ISC *Wald χ* ^2^ = 26.9, 60.8, respectively, *df* = 1, *p*<<0.001). For BL-FACES, unlike other tasks, including motion regressors seemed to hinder agreement between complexity and activation, and the best combination involved only detrending, regressing out global mean, and high-pass filtering (*d-bcw-fhi* AUC-T, AUC-ISC *Wald χ* ^2^ = 30.2, 34.1, respectively, *df* = 1, *p*<<0.001).

**Figure 7:**
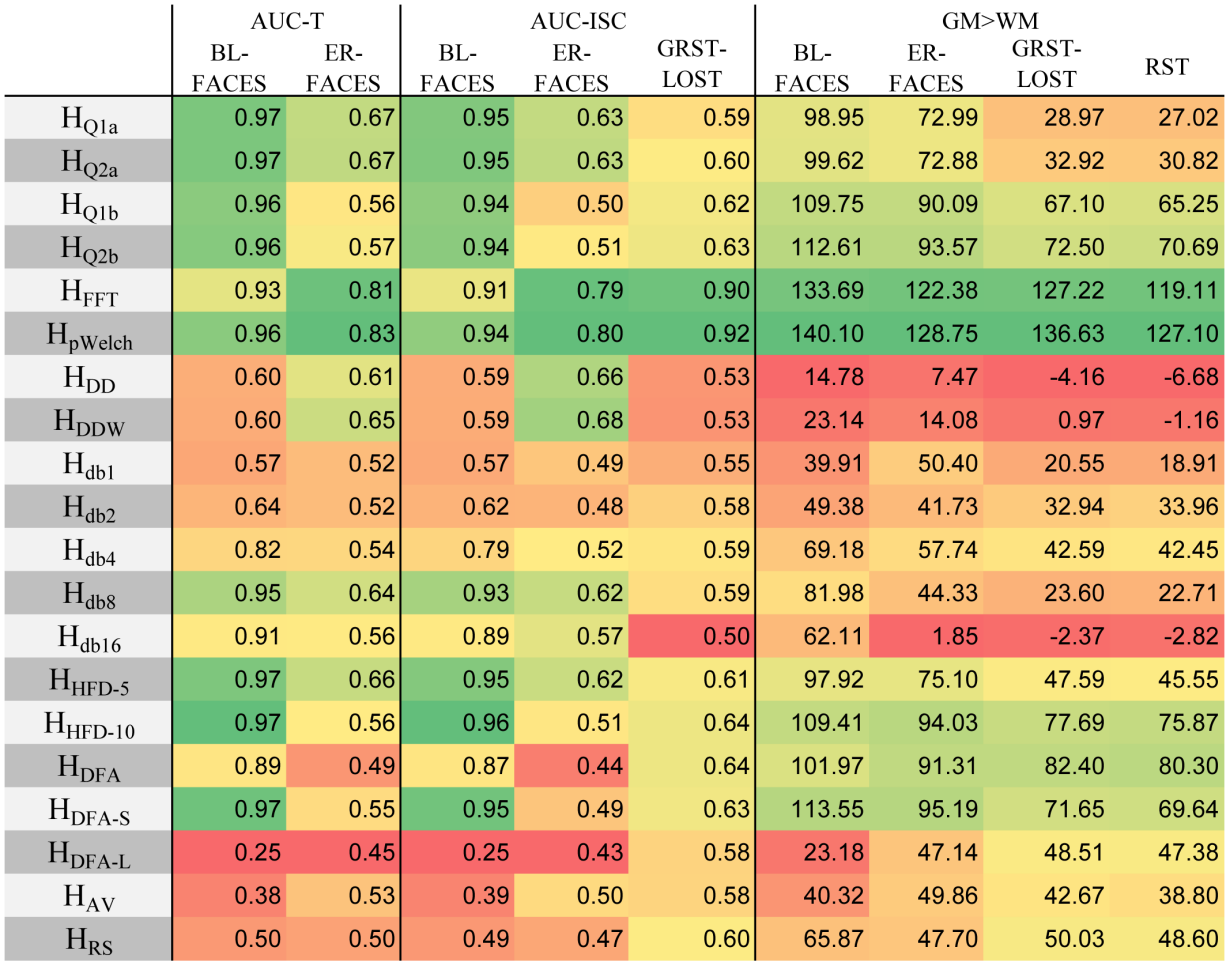
Sensitivity of Hurst estimates to activation and tissue type. The presented measures of sensitivity of Hurst estimates to activation and tissue type show that power-spectrum based measures are the most sensitive. The AUC-T values were derived from ROC curves constructed using top 1% of t-values of *task>rest* contrast. The AUC-ISC values were derived from ROC curves constructed using top 1% of z-transformed inter-subject correlations (ISC). The GM>WM values were derived from differences in Hurst estimates for grey and white matter voxels. The signal processing pipeline applied to the time series was *d-m-bcw-fhi* (see text). The values presented are averages across subjects (FACES: N = 22, RST & GRST-LOST: N = 11).

Since the second order motion correction did not significantly improve AUC or GM>WM contrast, we chose to focus on the *d-m-bcw-fhi* combination for rating the measures. We ranked the performance of each measure in terms of sensitivity to activation measured by AUC and to tissue type measured by GM>WM contrast (**Figure 7**). AUC-T and AUC-ISC ranks were averaged for the FACES tasks to avoid double-counting. For sensitivity to activation, in order from best to worst, the Hurst estimates ranked: *H_pWelch_, H_FFT_, H_HFD10_, H_Q2a_, H_HFD5_, H_Q1a_, H_Q2b_, H_DFA-S_, H_Q1b_, H_db8_, H_DFA_, H_db4_, H_DD_, H_DDW_, H_db16_, H_RS_, H_db2_, H_AV_, H_db1_, H_DFA-L_*. Similarly, for sensitivity to tissue type, the estimates ranked: *H_pWelch_, H_FFT_, H_HFD10_, H_DFA-S_, H_Q2b_, H_DFA_, H_Q1b_, H_HFD5_, H_RS_, H_Q2a_, H_db4_, H_Q1a_, H_DFA-L_, H_AV_, H_db2_, H_db8_, H_db1_, H_db16_, H_DDW_, H_DD_*.

**Figure 8:**
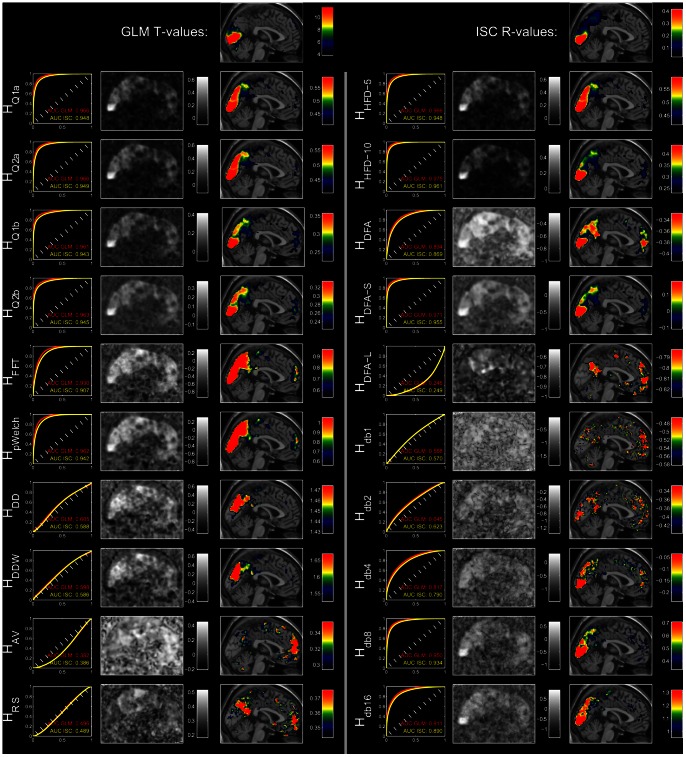
Hurst parameter estimates for the block faces task closely follow activation shown in the top row of the figure. The top left pane reflects activation (*t*-scores) for the contrast of *task>rest*, which in this case is presentation of emotionally valent faces vs. a fixation cross. The top right pane illustrates inter-subject correlations; scores presented as *r*-values for illustrative purposes. Rows below the first one show, for each measure: the ROC curves for agreement between complexity and activation with corresponding AUC values, the sagittal middle slice showing the distribution of *H*-values within and outside the brain, and the same slice overlaid with the heat map of highest *H*-values. All heat maps present the top 10% of the values in the entire image.

The performance of each of the measures is illustrated in **Figure 8**, which shows a close relation between activation and complexity in the BL-FACES task. The ROC curves constructed using the top 1% of GLM *t*- or ISC *r*-values show strong agreement for H_Q*_, *H_FFT_*, *H_pWelch_*, H_HFD*__,_ *H_DFA_*, *H_DFA-S_*, *H_db8_* and *H_db16_*. The AUC values are the same as those presented in **Figure 7**. The middle columns of the left and right panel of the figure qualitatively illustrate the distribution of complexity values in the brain, with *H_pWelch_* and *H_FFT_* showing the most discernible edges of the brain. The right columns show a cross-section through the middle slice of the top 10% of the values of the corresponding image. The top row shows that the bulk of the activation to the *task>rest* contrast resides in the visual cortex and that the inter-subject correlation also picked up the co-activation in the visual cortex across subjects, though in this case high *r*-values extended further into the midbrain. Most of the Hurst estimates picked out the visual and the prefrontal areas; in fact, the complexity measures accentuated these areas much more strongly than the activation maps themselves. For the complexity measures, these cross-sections also explain the AUC values, especially the low ones: some measures such as *H_AV_* and *H_DFA-L_* fail to adequately cover the visual areas and instead show higher *H*-values in the prefrontal regions, some (H_db*_) are grainy, and others (*H_DDW_, H_DD_, H_RS_*) fall somewhere in between.

### 3.5. Relation to Emotional Content (Arousal and Valence)

**Figure 9:**
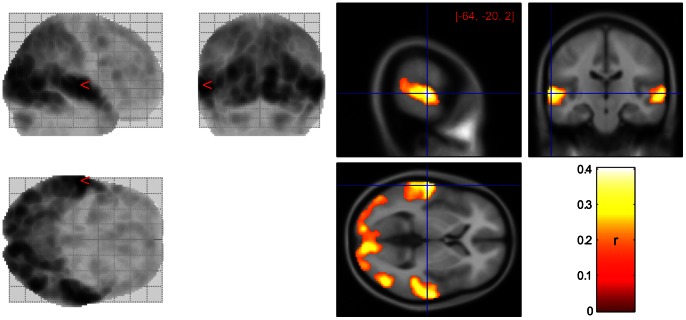
Inter-subject correlation across eleven participants for GRST-LOST task shows that most activation is restricted to areas involved in audiovisual processing. The glass brain (left) shows distribution of ISCs throughout the brain, while the heat map (right) outlines the areas deemed active at *r*>0.15. Though the activation appears symmetric, the maximally activated voxel is located on the left (MNI = −64, −20, 2).

**Figure 10:**
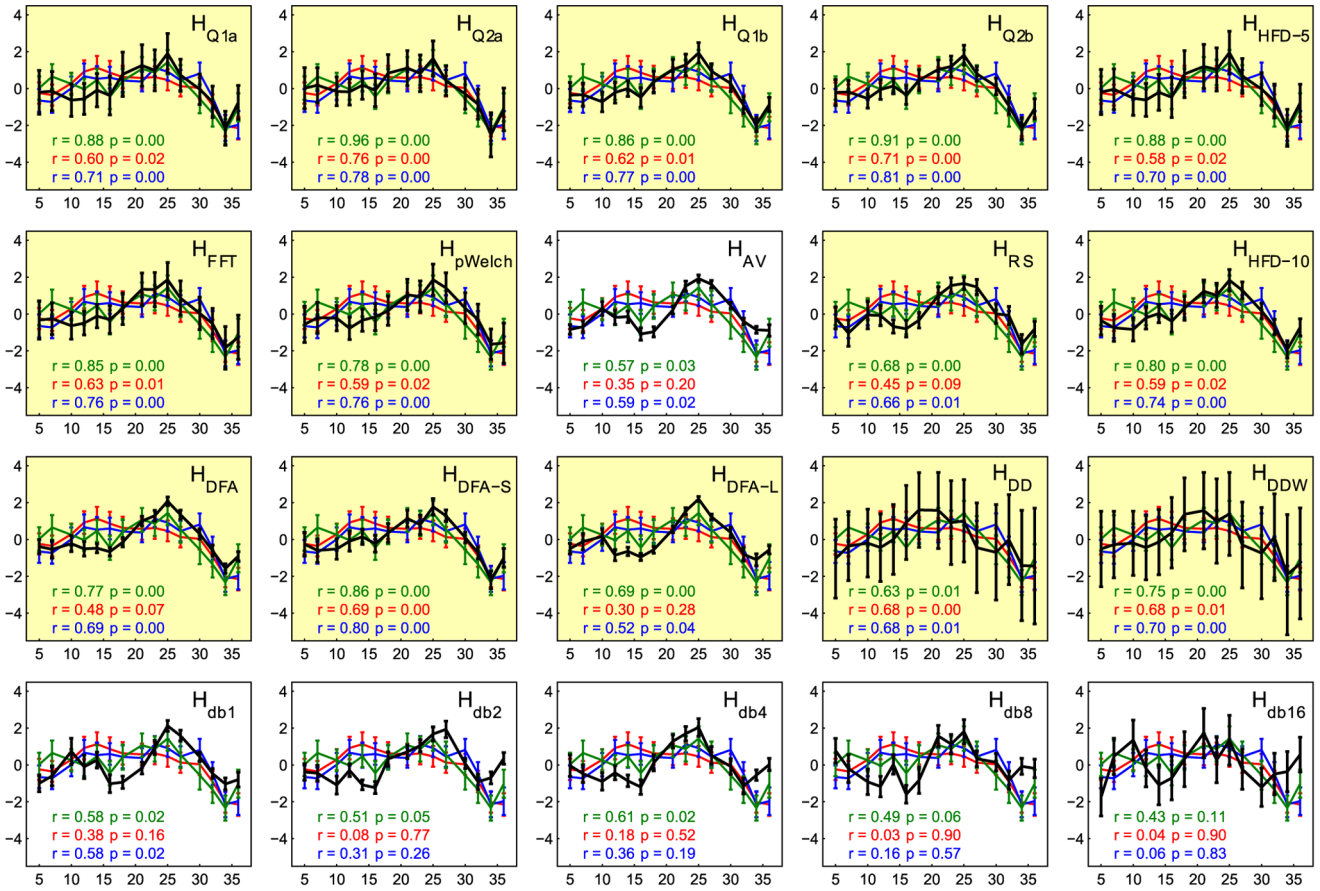
Variation in Hurst exponent estimates (black) correlates with arousal (red), valence (blue), and ISC-derived activation (green) throughout the GRST-LOST task. All estimates were computed over fifteen 10-minute windows evenly distributed throughout the task with 77% overlap, with the window centers plotted on the x-axis and z-scores of normalized variables on the y-axis. Error bars represent standard errors across the 11 participants. For arousal and valence, the positive values represent segments of the show rated as more calm and pleasant, while negative ones represent excited and unpleasant. The self-report measures were highly correlated (*r* = 0.92, *p*<<0.001). Highlighted plots represent complexity measures that correlated with either valence, arousal, or activation at *p*<0.01 level.

Not only did the complexity measures show high sensitivity to activation in tasks in their entirety, but the complexity also fluctuated with the emotional content of the LOST episode. We used the inter-subject correlation across the entire GRST-LOST task to identify regions engaged in passive viewing of the TV-episode, which, unsurprisingly, were well-associated with audiovisual processing (**Figure 9**). We then extracted the time series from the top 5% of the values in the brain (r>0.15), subdivided them into fifteen overlapping 10-min segments, and computed both complexity and average arousal and valence ratings of each segment. Complexity was strongly associated with the change in the emotional content for most of the Hurst estimates, with a slightly stronger association with the valence (**Figure 10**). Although *H_AV_*, *H_DFA-L_*, and H_db*_ preserved the overall shape of the response, their relation to self-report measures was either marginally or non significant. The second-derivative based estimates H_DD*_ showed large variance in estimates across subjects, deeming these measures less robust.

In addition to reflecting emotional content, Hurst estimates also varied with the average inter-subject correlation computed over the same 10-min segments (**Figure 10**) within the areas identified as active (**Figure 9**). The majority of measures showed strong correlation between *H* and ISC, with *H_AV_* and H_db*_ being least responsive in this context as well.

### 3.6. Optimal Task Length

**Figure 11:**
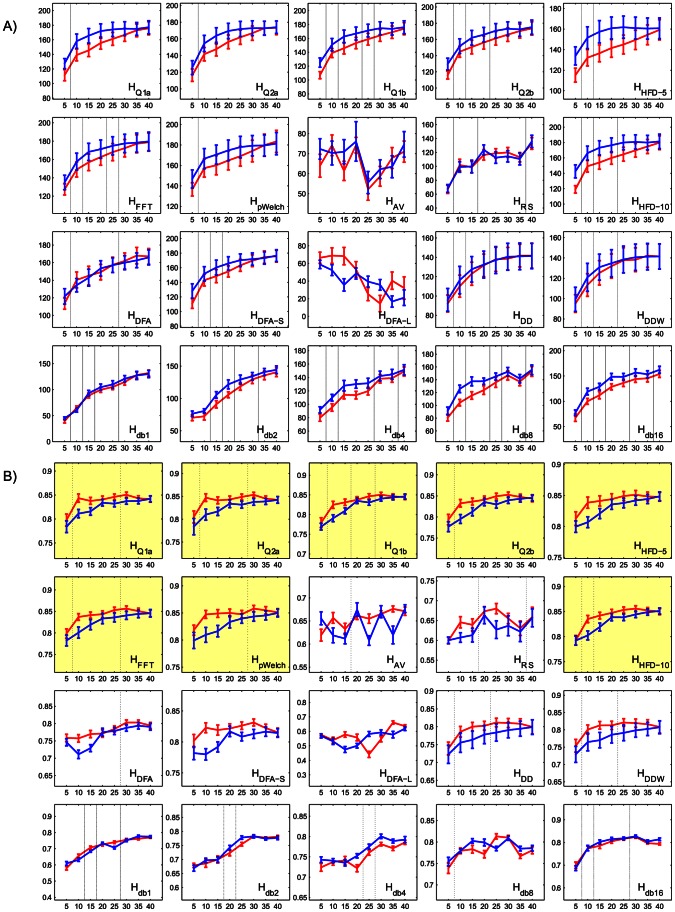
Mean (and standard error across 11 subjects) (A) GM>WM contrast (T-values on y-axis) and (B) overlap with activation for top 10% of the values (AUC on y-axis) increase for most of the Hurst estimates as the length of the time series (x-axis, minutes) increases. The two lines represent windows that start at the beginning (red) and the end (blue) of the task. Vertical lines between pairs of window sizes indicate that the subsequently larger window size significantly improves GM>WM contrast (A) or AUC (B) as compared to the smaller one for both forward and backward counting windows (p<0.025 each). The highlighted plots in (B) indicate measures that attained an AUC of 0.85 or more.

One of the most often raised concerns regarding the computation of complexity measures is the number of points in a time series necessary for a robust estimate of the Hurst exponent. The unusually long for fMRI GRST-LOST task was designed, in part, to address this issue. **Figure 11** shows the increase in grey-to-white matter contrast and AUC for time series from five to forty minutes in length, starting from the beginning and the end of the task. For the majority of measures, both GM>WM contrast and AUC increase monotonically with the number of points. Most of the measures plateau past the 20-min mark, suggesting that increasing time series length past a certain point ceases to improve estimation of complexity. The largest yield in performance per length of task occurs around the 10-minute mark, which means that in order to properly estimate *H*, the fMRI task has to be at least that long.

### 3.7. Scanner Differences (fMRI data)

**Figure 12:**
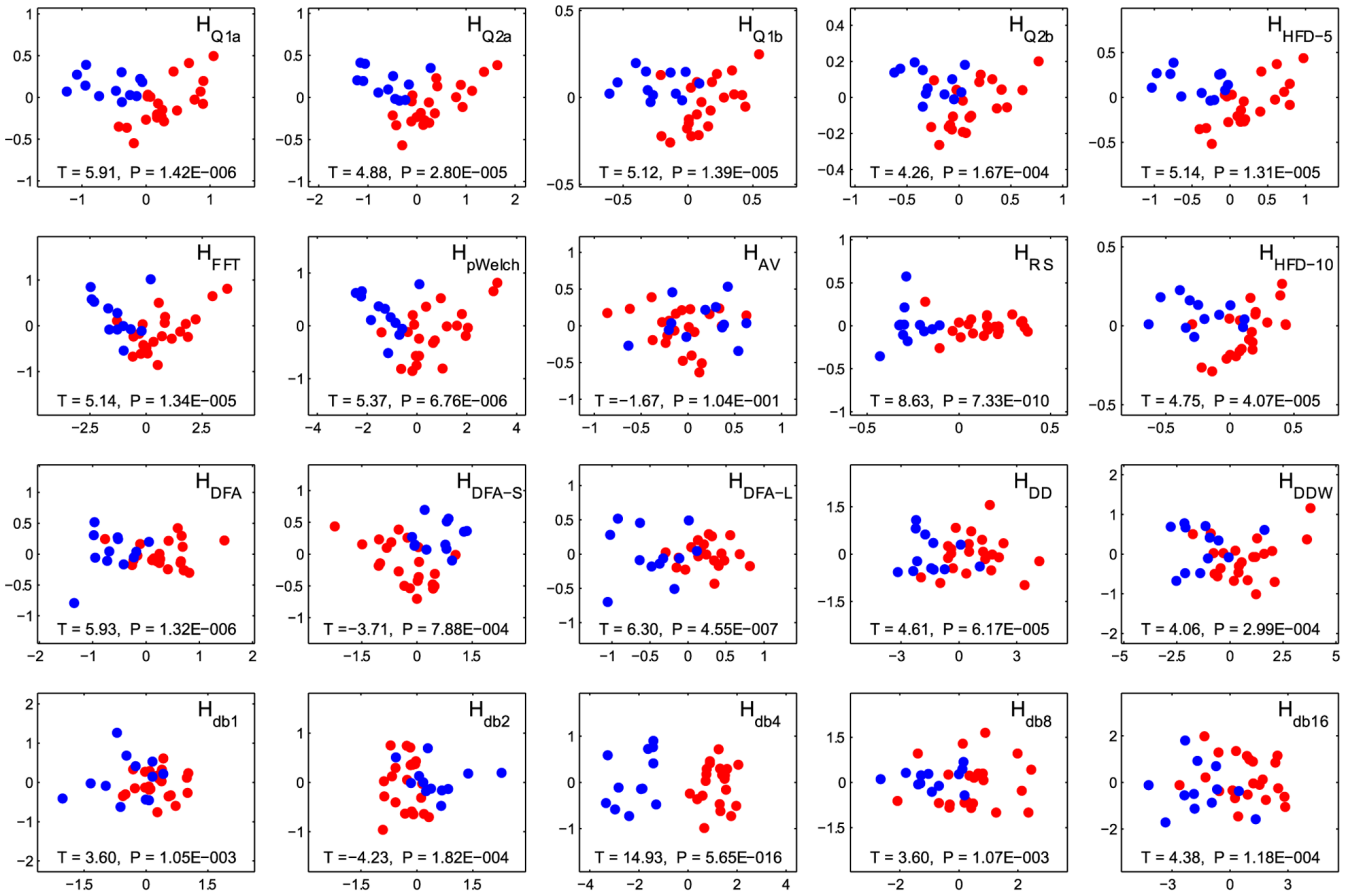
Strong scanner differences illustrated through principal components analysis. The red and blue points correspond to sets of subjects scanned on different scanners with the same task (BL-ANT). Each of the plots show the distribution of subjects along the first (x-axis, arbitrary units) and second (y-axis, arbitrary units) principal components of a matrix of Hurst region averages by subject. Each of the Hurst estimates was computed for the *d-m-bcw-fhi* processed average time series of every region of WFU pickAtlas. The *t*- and *p*-values represent differences between scanners along the first principal component (x-axis).

Given the increasing interest in shared, multi-institutional, data sets, as well as the development of neurodiagnostics, the degree to which complexity measures are sensitive to scanner differences is important. The BL-ANT task was run on two 3T scanners at the same institution – N = 22 on a Siemens and N = 12 on a Philips – using the same acquisition parameters. Activation areas and levels were equivalent across scanners [26]. Although we used independent populations for the different scanners, the groups were equivalent in terms of age (*t* = 1.98, *p* = 0.06, *df* = 32, mean age diff = 4.5 yrs), sex (*t* = 1.17, *p* = 0.25, *df* = 32), handedness (*t* = 0.64, *p* = 0.53, *df* = 32), or trait anxiety (*t* = 0.62, *p* = 0.54, *df* = 32). Despite this, principal component analysis (PCA) of Hurst exponents clearly separated the two groups across the first principal component (**Figure 12**, x-axis). The differences were highly significant for all estimates except *H_AV_*. The principal components contained information about Hurst estimates derived from average time series from every region of the WFU pickAtlas [47], [48] for each participant.

## Discussion

### 4.1. Relation to Activation

One of the claims made in the past was that the fractal nature of the fMRI signal is connected to activation [20], but the question remains as to whether results obtained from complexity versus statistical measures are different only in sensitivity or also in kind. Task designs are usually optimized to elicit maximal activation as determined by applying a general linear model to the data. This means that regions of the brain that are active irrespective of the task are ignored (e.g. maintenance of homeostasis). Because scale-invariance measures require hundreds of points, deriving condition-specific assessments of scale-invariance in fMRI is currently not viable. This discrepancy in the approach makes conceptualizing the relationship between the two types of analysis nontrivial. However, there are several avenues we can take in order to make inferences about this relation.

We chose to validate against a simple paradigm that used well-established stimuli (i.e. Ekman and Karolinska faces), so that the activated brain areas would be previously established. For these tasks (BL-FACES and ER-FACES), the bulk of activity is associated with processing of a cohort of emotional faces picked out by the *task>rest* contrast that compares it to a null condition that simply shows a fixation cross. To identify active voxels common to every subject, we correlated time series across pairs of subjects (**Figure 8**; results for ER-FACES very similar, not shown). These heat maps give different weighting to brain activity, stressing the voxels that fluctuated together more than those showing greatest difference in response to the stimuli.

The heat maps of the Hurst estimates accentuate activity in the visual cortex much more strongly than the activation maps themselves, indicating that they are, in a sense, more sensitive. This is consistent with the previous publications [19], [20] that imaged four slices along the calcarine sulcus and showed that active voxels exhibit distinctly different behavior across nonlinear measures (such as Hurst exponent and its multifractal counterpart) even when applied to the residuals of GLM regression. However, the Hurst estimate maps extend beyond activation maps into the medial surfaces of precuneus, posterior parietal cortex, and posterior cingulate gyrus and also show a distinct “hot spot” in the medial prefrontal cortex. This indicates that although Hurst measures may be more sensitive to some aspects of brain activity, they certainly do not show one-to-one correspondence to “activation” defined by the GLM, as referred to by most of the fMRI literature.

### 4.2. Relation to Emotional Content (Arousal and Valence)

Given the nature of the show, the participants’ ratings of arousal and valence of the content were highly correlated. Across the fifteen windows, the correlation between arousal and inverted valence was very high (*r* = 0.92, *p*<<0.001), which meant that most exciting clips were unpleasant and the less exciting ones were found to be pleasant. The positive correlation to complexity suggests that active regions had higher complexity when processing the more calm/pleasant content rather than the exciting/unpleasant one. Given the positive correlation to ISC, periods of higher complexity also had higher synchronized (across subjects) activity.

Although both self-report measures correlated with ISC-based activation (*r* = −0.73, *p* = 0.002 for arousal; *r* = −0.70, *p* = 0.004 for inverted valence), they showed less variation with complexity than ISC potentially due to the fact that they are discrete, limited in range, and prone to inaccurate representation of the true nature of emotional content of the show as the scenes are separated by a considerable amount of time and other emotional content.

### 4.3. Scanner Differences

Because the first principal component contained information about the whole brain divided into regions according to the WFU PickAtlas [47], [48] for each person, it is unlikely that group differences arose due to local differences in brain activity. Furthermore, the differences in Hurst exponents between groups are very strong, arguably much stronger than between any two random samples of healthy human participants. This serves as an indication that the main variable driving the effect is the choice of scanner. Although the scan parameters were the same (TR, TE, flip angle, number of slices, gap, FOV, reconstruction matrix), the implicit differences between scanners contributing to background noise were strong enough to be picked up by the Hurst estimates and clearly separate a uniform group of people. The exact causes that give rise to differences in properties of time series across scanners require a more rigorous investigation, but overall, this comparison shows that while combining datasets across scanners may in some cases be acceptable for standard linear model analyses, it is not for complexity investigations, unless the scanner differences are explicitly modeled or corrected for.

## Conclusions

### 5.1

**Figure 13:**
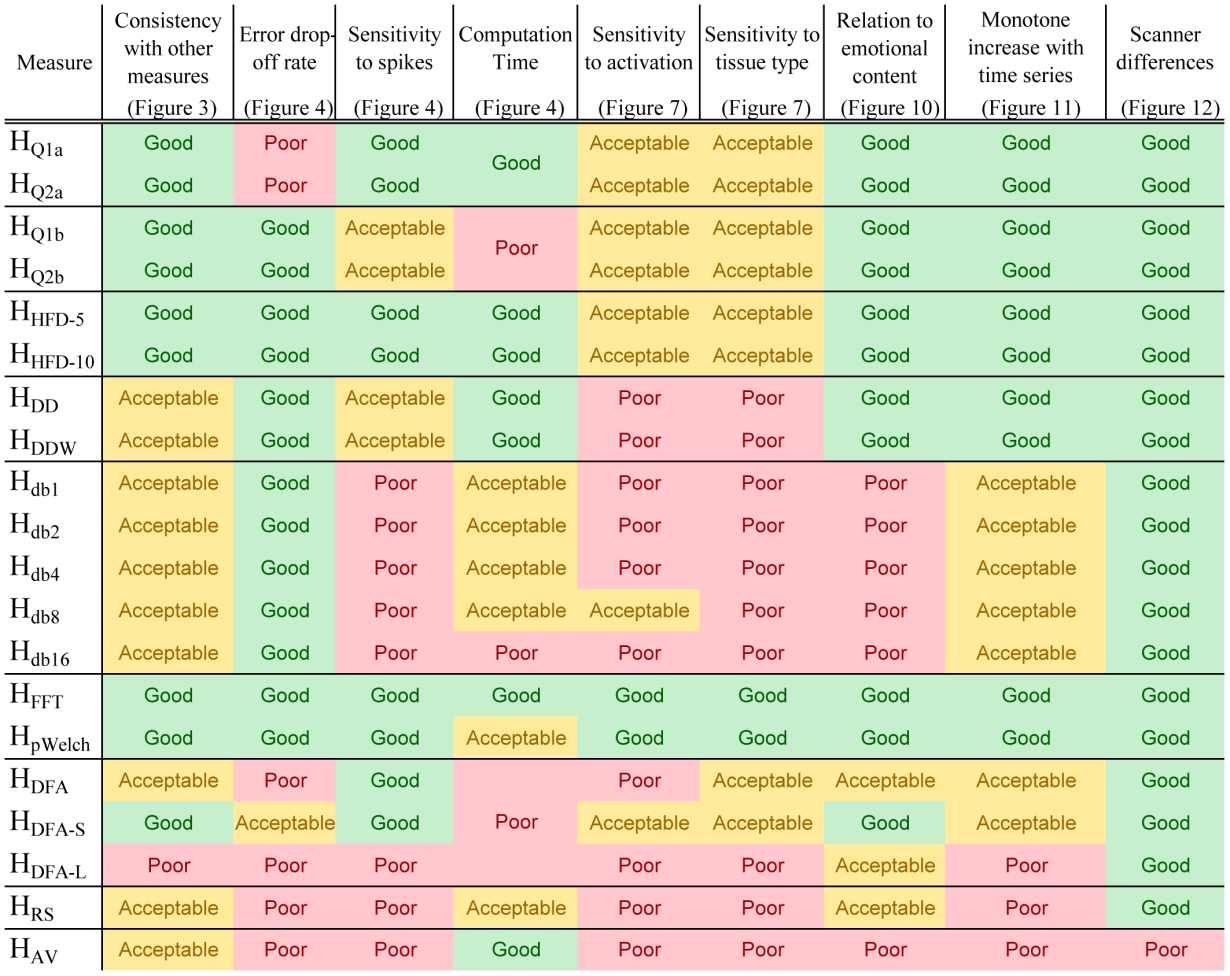
Summary of performance of Hurst estimates. Summary of measure comparisons highlights *H_FFT_*, *H_pWelch_*, and H_HFD*_ as the most reliable methods overall.

The comparisons based on simulated and experimental data show that complexity measures are highly correlated to each other and indeed measure the same quantity, but with differing sensitivity and susceptibility to artifacts (**Figure 13**). The poorest performing measures overall were H_db*_, H_DFA*_ (except *H_DFA-S_*), *H_AV_*, and *H_RS_*. Daubechies wavelet based computations (H_db*_) have long computation times, are not sensitive to spikes, and show poor sensitivity to activation, tissue type, and emotional content; for these Daubechies wavelet based estimates the overall performance increases with the wavelet order up to a point (*H_db8_*), and then deteriorates. *H_RS_* and *H_AV_*, performed poorly across the board. In terms of image contrast, overlap with activation, and group differences, H_DFA*_ performed poorly as well, with *H_DFA-S_* outperforming *H_DFA_* and *H_DFA-L_*, suggesting that the bulk of useful information is found at shorter lags. The computation times were also very high for this set of measures. The H_Q*b_ measures are naturally very similar to H_Q*a_, but are the longest to compute, with the error drop-off rate as the only distinguishable characteristic.

The most consistently successful measures were the power-spectrum based measures *H_FFT_* and *H_pWelch_*, with the latter slightly outperforming the former while taking much longer to compute. H_HFD*_ did not stand out in either a positive or a negative way, but performed consistently well across all the comparisons, ranking second overall behind the power-spectrum based measures, with *H_HFD-10_* being slightly better than *H_HFD-5_*. H_Q*a_ took the third place, having failed only one comparison.

### 5.2. The Bottom Line

The results outlined above recommend certain steps for estimating complexity of fMRI time series. First, it is crucial to either collect data using a single scanner or control for this effect. Second, it appears that detrending, regressing out the global mean, and excluding low frequencies improves agreement between complexity and activation. First order motion correction also has a small, but significant improvement on tissue contrast. Third, given the typical two-second acquisition time for fMRI, the tasks should last about 20 and definitely more than 10 minutes. Finally, the best measures to use are either the power-spectrum based ones (*H_FFT_* or *H_pWelch_*) on a restricted frequency range (above ∼0.01 Hz), or H_HFD*_ or H_Q*a_ on filtered data.

Although the comparisons of numerical implementations of various algorithms over the simulated data give a “clean” comparison, the real usefulness of Hurst estimates is, however, assessed by their relation to an interpretable quantity, such as activation or tissue type. The better estimates, as outlined in this paper, are more sensitive to some aspects of brain activity than linear regression and correlation approaches. Despite the potential utility of complexity in neuroimaging, the underlying neuronal and BOLD features that give rise to the differences in Hurst estimates are not mechanistically well understood, and future investigation will be necessary to address this important question.
